# Utility of C-reactive protein on the fourth postoperative day to detect complications beyond anastomotic dehiscence

**DOI:** 10.1007/s00384-025-04912-y

**Published:** 2025-05-20

**Authors:** David Ortiz-López, Joaquín Marchena-Gómez, Yurena Sosa-Quesada, Manuel Artiles-Armas, Eva María Nogués-Ramia, Beatriz Arencibia-Pérez, Julia María Gil-García, Cristina Roque-Castellano

**Affiliations:** 1 Department of General and Digestive Surgery, University Hospital of Gran Canaria Doctor Negrín, Barranco de la Ballena s/n, Las Palmas de Gran Canaria, Canary Islands 35010 Spain; 2 University of Las Palmas de Gran Canaria, Las Palmas de Gran Canaria, Canary Islands Spain

**Keywords:** Colorectal cancer, C-reactive protein, Postoperative complications, Anastomotic dehiscence, Comprehensive complication index

## Abstract

**Purpose:**

Postoperative complications can affect recovery after colorectal cancer surgery. Elevated C-reactive protein (CRP) levels have been studied as a predictor of anastomotic dehiscence, but evidence regarding its association with overall complications is limited. This study aimed to explore the link between CRP levels on the fourth postoperative day and overall postoperative complications using the comprehensive complication index (CCI).

**Methods:**

The observational study included 935 patients who underwent colorectal cancer surgery between 2015 and 2022. Patients were categorized into three groups: no complications, complications excluding dehiscence, and complications with dehiscence. The relationship between CRP levels and postoperative complications was analyzed, and the optimal CRP cutoff point was determined.

**Results:**

The median CRP values were 34.3 (20.4–54.0) mg/L in the group with no complications, 69.9 (43.2–112.9) mg/L in the group with complications excluding dehiscence, and 167.6 (69.7–239.5) mg/L in patients with dehiscence. A significant correlation between CRP levels and postoperative complications was found (*p* < 0.001). Based on the identified cutoff points, CRP levels above 58 mg/L suggest the presence of any complication, including dehiscence. Levels between 42 and 58 mg/L suggest complications excluding dehiscence, and levels below 42 mg/L strongly exclude complications, with a negative predictive value of 82%.

**Conclusions:**

Elevated CRP on postoperative day 4 is associated with overall postoperative complications, not just dehiscence. A positive correlation exists between CCI score and CRP levels. A CRP value < 42 mg/L on day 4 allows clinicians to reliably exclude the presence of any complication.

**Supplementary information:**

The online version contains supplementary material available at 10.1007/s00384-025-04912-y.

## Introduction

Colorectal cancer is the second leading cause of cancer-related mortality, with an overall 5-year survival rate of 64% [1]. The prognosis of the disease is better in patients diagnosed at early stages and those who can undergo surgery [2].

Colorectal cancer surgery carries a non-negligible morbidity and mortality rate which may impact long-term survival, although there are contradictory findings in the scientific literature [3–5]. Therefore, it is important to minimize the occurrence of postoperative complications and their impact on the patient.

There is considerable scientific evidence regarding the role of postoperative analytical parameters in predicting the onset of postoperative complications [6]. Primarily, the relationship between C-reactive protein (CRP) levels and anastomotic dehiscence has been studied [7].

However, there is limited evidence regarding whether changes in CRP are associated with the occurrence of overall postoperative complications, in addition to anastomotic dehiscence. The studies that investigate this typically rely on the Clavien-Dindo classification [8]. Fewer data are available on this relationship using the comprehensive complication index (CCI) [9], which may be more useful for assessing the overall severity of all postoperative complications [10].

This study aimed to analyze the relationship between elevated CRP levels on the fourth postoperative day (CRP-4POD) and the occurrence of postoperative complications according to the CCI classification. Additionally, we aimed to compare the elevation of CRP-4POD levels based on whether the postoperative complication was anastomotic dehiscence or another complication unrelated to dehiscence.

## Methods

### Study design

This was a retrospective, observational, and longitudinal study that included 935 patients who underwent surgery for colorectal cancer at our institution between January 2015 and December 2022. All patients diagnosed with colorectal cancer were included, regardless of stage, provided they were treated with curative intention. Patients who underwent emergency surgery for complicated colorectal cancer were excluded.

This study was performed in line with the principles of the Declaration of Helsinki [11] and reported according to the STROBE guidelines [12]. Approval was granted by the Clinical Ethics and Research Committee of the institution (2020–279-1).

### Patient management

The diagnosis was made through colonoscopy and biopsy, while staging was performed using thoracoabdominal computed tomography (CT) and pelvic magnetic resonance imaging (MRI) for patients diagnosed with rectal cancer. The stage was determined according to the 8 th edition of the AJCC TNM classification [13].

Patients were admitted the day before surgery, and a baseline analytical assessment including a complete blood count was performed. All patients underwent mechanical bowel preparation with oral antibiotics.

Surgery was performed by specialist colorectal surgeons, with most procedures being minimally invasive (laparoscopic and robotic surgery). The anastomosis technique was carried out using mechanical suturing devices.

Postoperatively, CRP levels were measured on the fourth day after surgery in all patients, and the results were expressed in mg/L.

The entire series was followed up for a median of 43.8 months (IQR, 23.4–69.4).

### Study groups

The sample was divided into three groups: patients without complications, patients with complications without anastomotic dehiscence, and patients with complications that included anastomotic dehiscence.

### Study variables

The following variables were collected:

- Demographic variables: age and sex.
- Comorbidity: Charlson Comorbidity Index. Comorbidity was stratified as low (0–2 points), moderate (3–4 points), and high (> 4 points).
- Tumor characteristics: location and tumor stage.
- Surgical variables: type of approach, conversion, type of surgery performed.
- Postoperative outcomes: postoperative complications with special attention to anastomotic dehiscence, reoperations, operative mortality, and postoperative length of stay (in days). Postoperative complications were scored using the comprehensive complication index (CCI), which is based on Clavien-Dindo and takes into account all adverse event. The score ranges from 0 (no complications) to 100 points (death). The CCI was subdivided into four groups: no complications (0 points), mild complications (1–26.1 points), moderate complications (26.2–42.2 points), and severe complications (> 42.3 points) [14]. The score was calculated using the following online tool: https://www.assessurgery.com/about_cci-calculator/.
- Operative mortality was defined as deaths occurring within the first 90 days after surgery or later if they were directly caused by a postoperative complication.

### Statistical analysis

The data were analyzed using the statistical software suite SPSS 29.0 for Windows (IBM Corporation, Armonk, NY, USA) and the software Jamovi 2.3 (The Jamovi Project, 2022).

First, a descriptive analysis of the sample was performed. Categorical variables were expressed as frequencies and percentages. Numeric variables were expressed as means (± standard deviation) (SD) or medians (interquartile range) (IQR), depending on whether the distribution followed a normal distribution. The Kolmogorov–Smirnov test was used to assess normality.

Next, a comparative study was conducted between the three study groups, analyzing the differences observed in CRP values and CCI scores for each group. The Kruskal–Wallis test was used for this, and the Spearman’s correlation coefficient was used to assess the relationship between CRP and CCI. Finally, the diagnostic performance of CRP in relation to CCI was analyzed in two scenarios: in the entire sample and in a sample of patients from which anastomotic dehiscences were excluded. Receiver operating characteristic (ROC) curves were constructed for each scenario to determine the respective cutoff points based on the Youden index [15], which represents the point of maximum sensitivity and specificity. Sensitivity, specificity, positive predictive value, and negative predictive value of CRP were then calculated for each scenario.

To evaluate the influence of the type of surgery (colonic vs rectal surgery) as a potential confounder in relation to postoperative CRP levels, a multivariable linear regression analysis was conducted. The model included complication type (no complications, complications without dehiscence, dehiscence) and was adjusted for surgery type (colonic vs rectal). The outcome variable was CRP-4POD. Variance inflation factors (VIFs) were computed to assess and exclude multicollinearity.

A significance level of *p* < 0.05 was considered.

## Results

The results of the descriptive analysis of the 935 patients included in the study are shown in Table 1.

**Table 1:** Demographic characteristics

Sex	
Male	585 (62.6%)
Female	350 (37.4%)
Age (median–IQR)	70 years (62.0–77.0)
Comorbidity (Charlson Index)	
Low	442 (47.27%)
Moderate	356 (38.07%)
High	137 (14.66%)
TNM stage	
I	219 (23.42%)
II	340 (36.37%)
III	308 (32.94%)
IV	68 (7.27%)
Tumor location	
Colon	669 (71.55%)
Rectum	266 (28.45%)
Specific location	
Right colon	306 (32.74%)
Left colon	81 (8.66%)
Sigmoid colon	212 (22.67%)
Upper rectum	75 (8.02%)
Medium rectum	128 (13.69%)
Low rectum	66 (7.05%)
Synchronic tumors	18 (1.93%)
Surgical technique	
Right colectomy	322 (34.44%)
Left colectomy	75 (8.02%)
Sigmoidectomy	196 (20.96%)
Upper anterior rectal resection	82 (8.77%)
Low anterior rectal resection	139 (14.87%)
Ultra-low anterior rectal resection	34 (3.64%)
Segmentary colectomy	40 (4.28%)
Total colectomy	18 (1.93%)
Combined resection	7 (0.74%)
Abdominoperineal amputation	22 (2.35%)
Surgical approach	
Open surgery	269 (28.77%)
Laparoscopic surgery	554 (59.25%)
Robotic surgery	112 (11.98%)
Conversion	
Yes	47 (7.06%)
No	619 (92.94%)
Length of stay	
Mean (± SD)	8.7 days (7.6)
Median (IQR)	6 days (5.0–9.3)
Postoperative complication	
No	627 (67.05%)
Yes	308 (32.95%)
CCI	
0	627 (67.05%)
1–26.2	152 (16.26%)
26.3–42.2	83 (8.88%)
> 42.2	73 (7.81%)
Type of complication	
Anastomotic dehiscence	51 (5.5%)
Hemorrhagic complication	65 (6.9%)
Adynamic ileus	118 (12.6%)
Surgical wound infection	35 (3.7%)
Intraabdominal collection	26 (2.78%)
Evisceration	12 (1.28%)
Central venous access related infection	39 (4.17%)
Cardiological complication	23 (2.46%)
Respiratory complication	37 (3.96%)
Nephro-urological complication	52 (5.56)
Other complication	65 (6.95%)
Readmission	38 (4.1%)
Reoperation	85 (9.1%)
Postoperative mortality	9 (1%)

Of the 935 patients, 308 (32.95%) had some type of complication, most of which were mild complications, with a CCI < 26.2 (152 patients, 16.26%).

Regarding the type of complication, 51 cases (5.5%) of anastomotic leakage were identified, along with 65 hemorrhagic complications (6.9%), of which 32 patients (3.4%) required red blood cell transfusion. Postoperative adynamic ileus was observed in 118 patients (12.6%), with 62 (6.6%) requiring parenteral nutrition. Additionally, 35 cases (3.7%) of surgical wound infections, 26 (2.78%) intra-abdominal collections, and 12 (1.28%) cases of evisceration were recorded.

Among non-abdominal complications, 39 patients (4.17%) developed infectious complications related to central venous access, 37 (3.96%) experienced respiratory complications, 23 (2.46%) presented with cardiological complications, and 52 patients (5.56%) had nephrourological complications.

A further 65 patients (6.95%) experienced other types of complications, including non-infectious surgical wound issues such as seromas or hematomas in 21 cases (2.2%), superficial infections of peripheral venous access in 15 cases (1.6%), ostomy-related problems in 12 patients (1.28%), and visceral perforation or intestinal obstruction requiring reoperation in 11 patients (1.18%).

Of the entire cohort, 38 patients (4.1%) were readmitted within 30 days of hospital discharge, 85 (9.1%) required reoperation due to complications related to the initial procedure, and 9 patients (1.0%) died within 90 days following surgery.

**Fig. 1:**
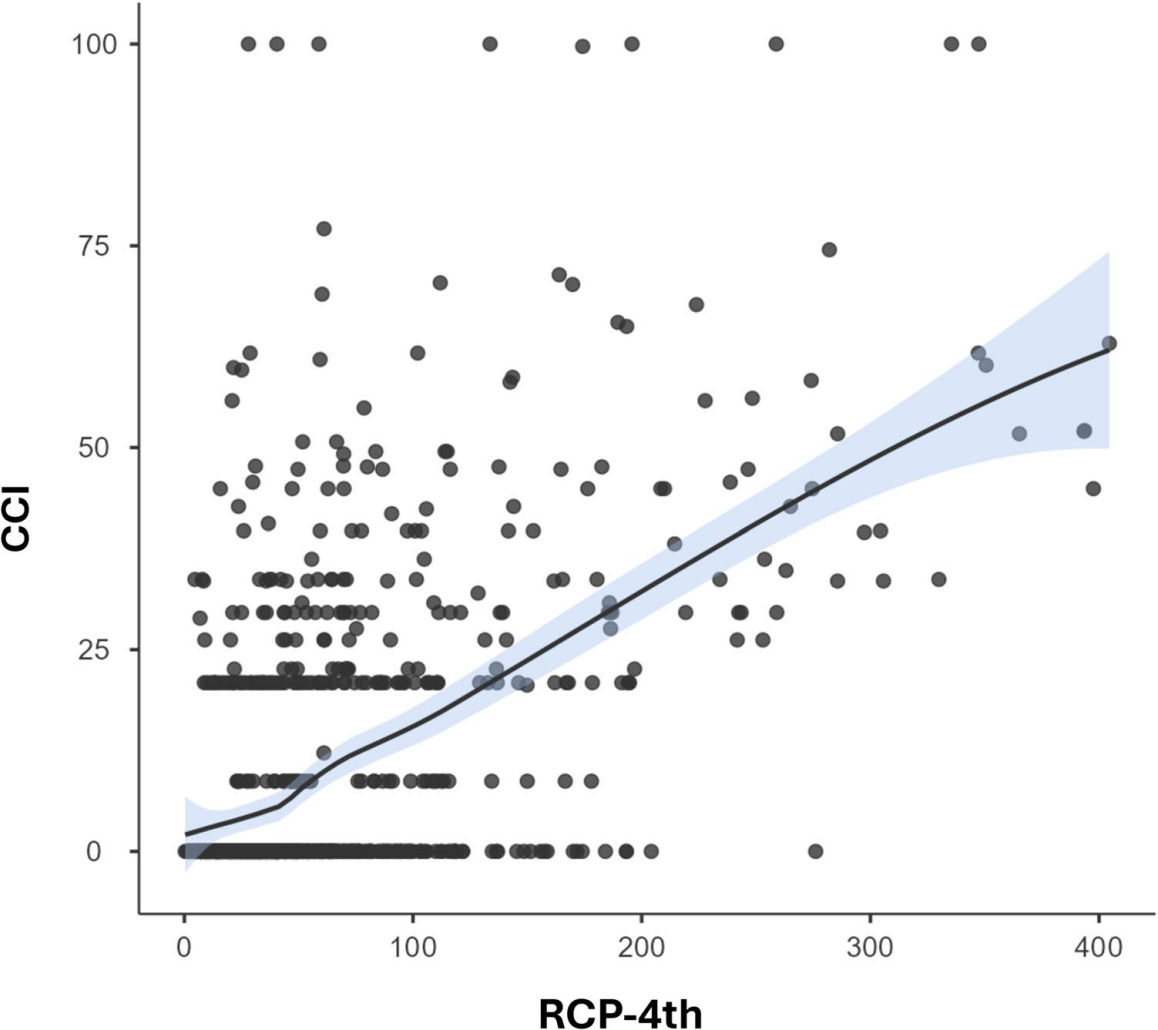
C-reactive protein on the fourth postoperative day correlation with comprehensive complication index (*p* < 0,001)

The mean CRP-4POD value for the entire sample was 62.28 (± 62.57) mg/L, with a median of 42.14 (IQR, 24.81–73.52) mg/L. The mean CCI score was 10.51 (± 18.77) points, with a median of 0.00 (IQR, 0.00–20.90) points. A very significant correlation was found between CRP levels on the fourth postoperative day and postoperative complications according to the CCI (*p* < 0.001) (Fig. 1).

**Fig. 2:**
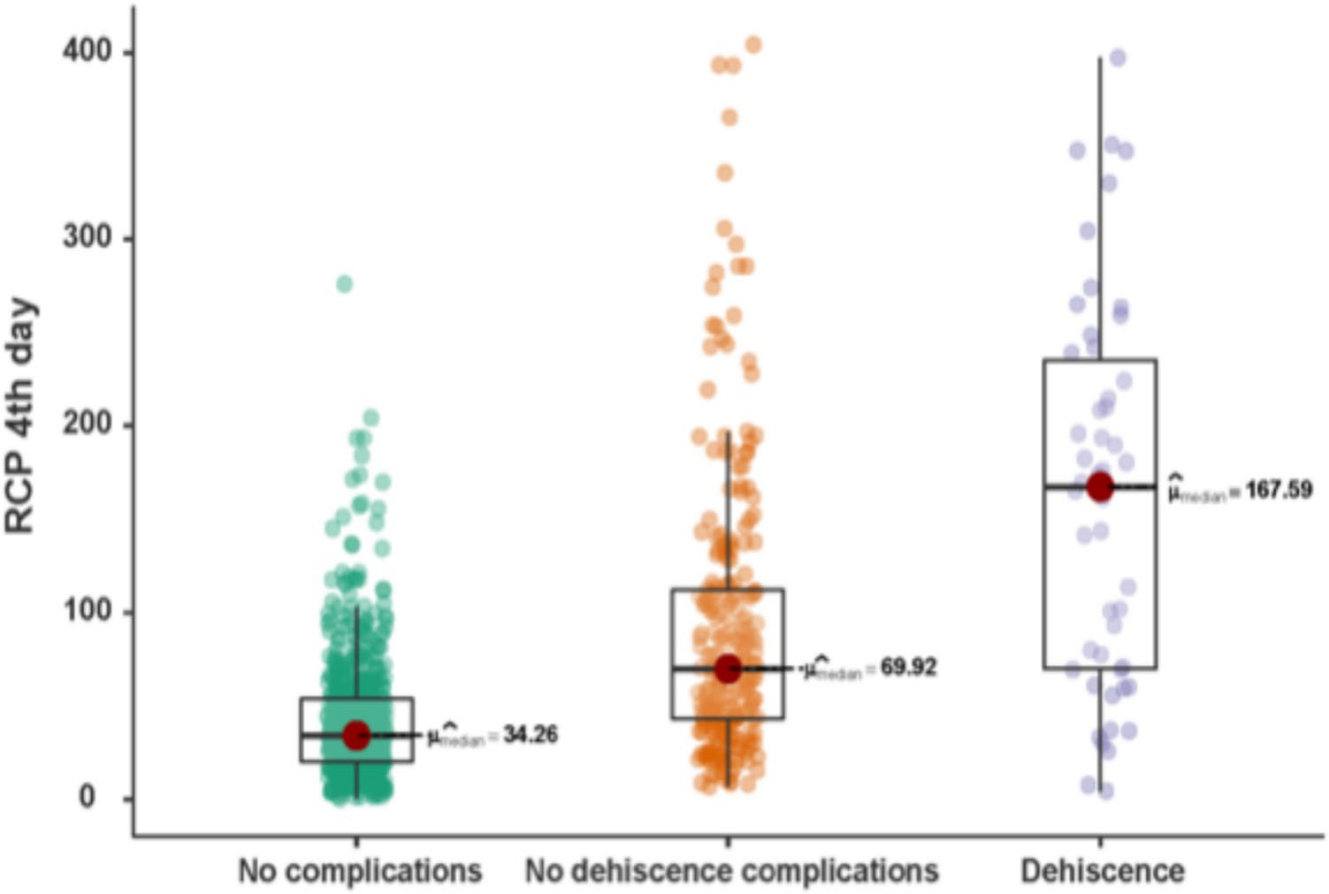
Differences in C-reactive protein between the no-complication group, the no-dehiscence complication group, and the dehiscence group (*p* < 0,001)

The median CRP-4POD values were 34.3 (20.4–54.0) mg/L for the 627 patients in the group with no complications, 69.9 (43.2–112.9) mg/L for the 257 patients in the group with complications excluding dehiscence, and 167.6 (69.7–239.5) mg/L for the 51 patients with dehiscence (Fig. 2). These differences were statistically significant (*p* < 0.001). Pairwise comparisons between the three groups for the CRP-4POD variable (no complications, complications excluding dehiscence, and dehiscence) also showed statistically significant differences for each comparison (*p* < 0.001).

In the multivariable analysis, the complication type variable (*p* < 0.001) behaved as an independent prognostic factor for CRP levels on postoperative day 4 (CRP-4POD) after adjustment for surgery type (colonic vs rectal) (*p* = 0.917). No collinearity was detected (VIF = 1.02 for both variables).

**Fig. 3:**
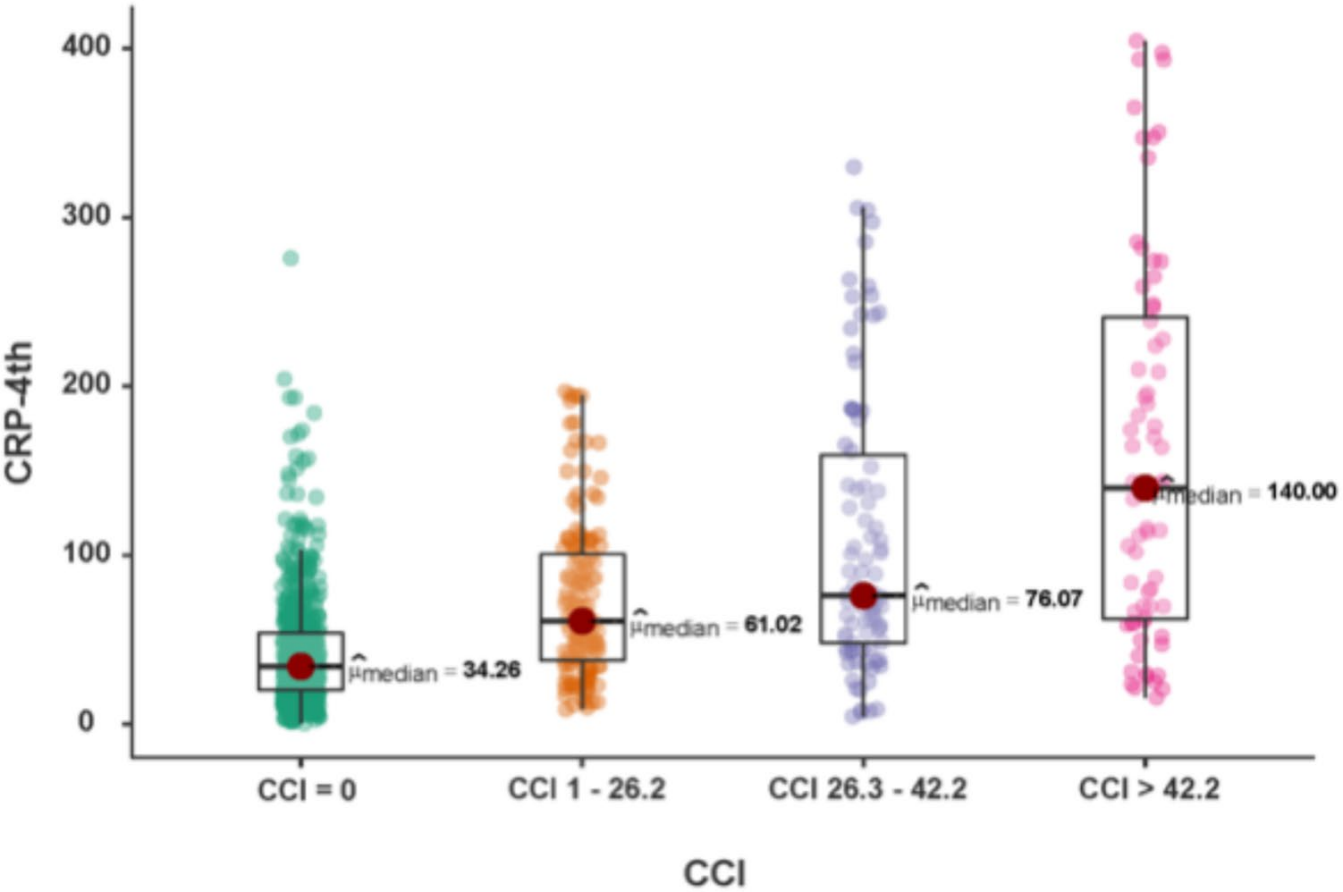
C-reactive protein comparison between CCI groups: no complications, mild complications, moderate complications, and severe complications (*p* < 0,001)

Likewise, when categorizing the CCI into no complications (0 points), mild (1–26.1 points), moderate (26.2–42.2 points), and severe (≥ 42.3 points) complications, statistically significant differences were observed in the CRP-4POD levels between all four categories (Fig. 3).

**Fig. 4:**
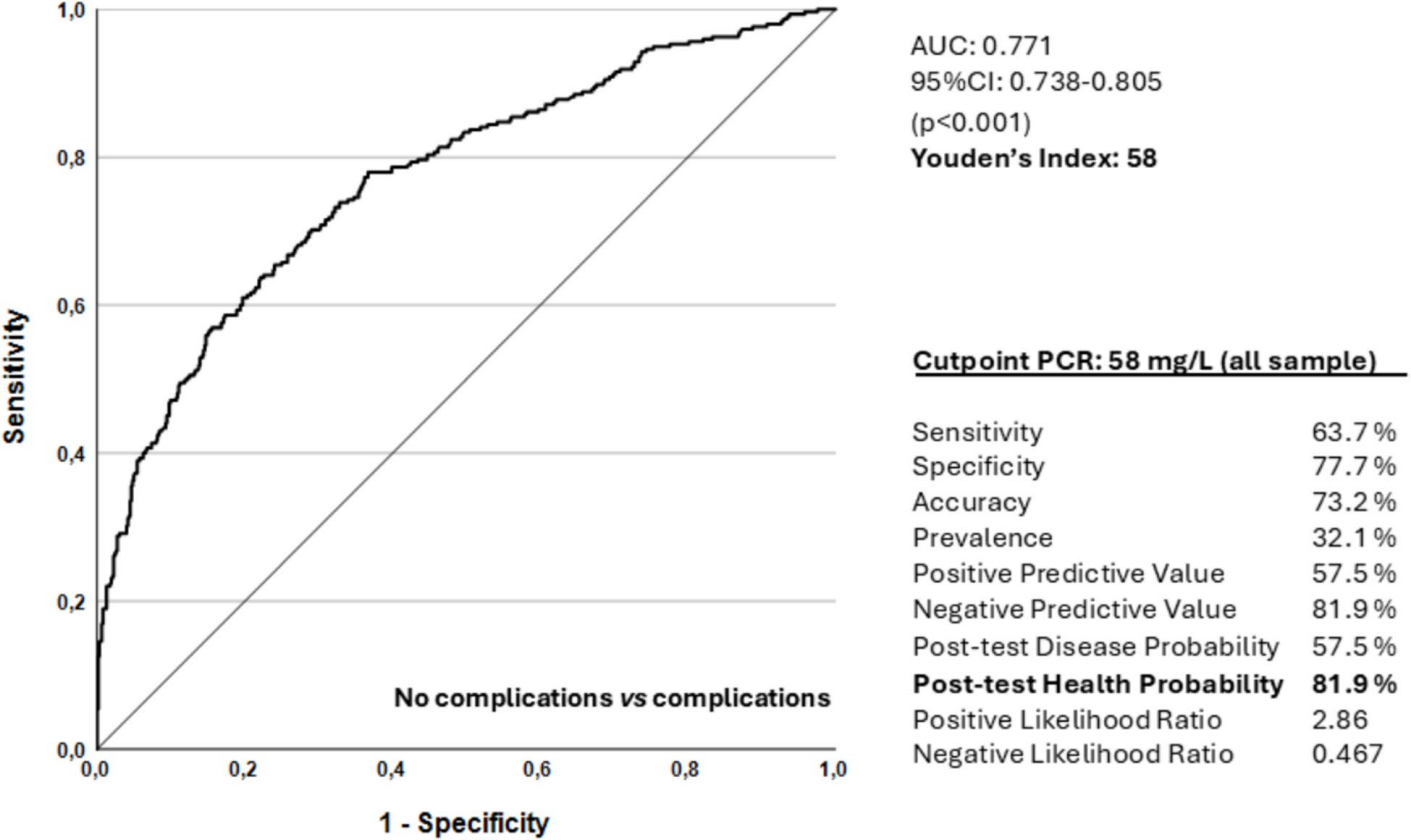
ROC curve and diagnostic parameters for the identification of patients with complications using a PCR cutoff value of 58 mg/L. AUC 0.771 (95% IC 0.738–0.805, *p* < 0.001)

Regarding the diagnostic ability of CRP-4POD to detect any type of complication (CCI = 0 vs CCI = 1), the results are shown in Fig. 4. According to the Youden index, the optimal cutoff point used in the analysis was 58 mg/L. This indicates that a CRP level above 60 mg/L on the fourth postoperative day should raise suspicion of some type of complication, including a potential anastomotic dehiscence. Levels below 60 mg/L exclude anastomotic dehiscence, as well as other complications, with a wide margin of safety.

**Fig. 5:**
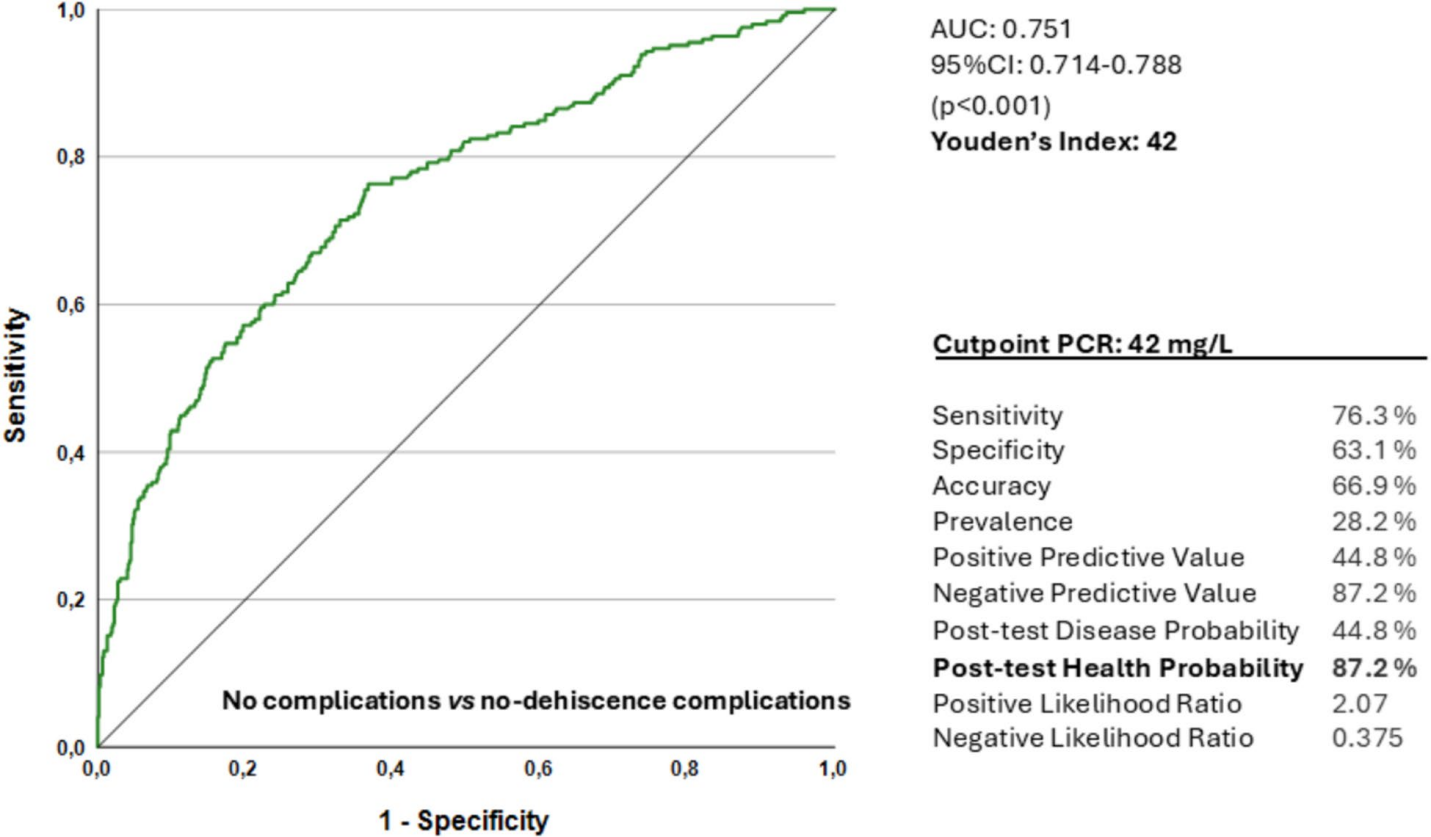
ROC curve and diagnostic parameters for the identification of patients with no dehiscence complications using a PCR cutoff value of 42 mg/L. AUC 0,751 (95% CI 0.714–0.788, *p* < 0.001)

Regarding the diagnostic ability of CRP-4POD to detect complications unrelated to anastomotic dehiscence (CCI = 0 vs CCI = 1, once patients with dehiscence were excluded from the sample), the results are shown in Fig. 5. In this case, Youden index indicated the optimal cutoff point at 42 mg/L. Therefore, a CRP level between 42 and 60 mg/L may indicate the presence of a complication other than anastomotic dehiscence, and levels below 40 mg/L exclude any type of complication with a wide margin of safety.

## Discussion

This study is in line with other publications regarding the relationship between CRP on the fourth postoperative day and anastomotic dehiscence [16–20]. It has also demonstrated its utility in predicting any type of postoperative complication, not just anastomotic dehiscence, as well as a positive correlation between CRP levels and the severity of complications, which are relatively underexplored in the scientific literature.

Among the results, we highlight the clear positive correlation between the absolute values of CCI and CRP, meaning that a higher CRP on the fourth postoperative day was associated with a higher postoperative CCI, and thus greater severity of postoperative complications. A statistically significant association was also observed between CRP-4POD and the CCI categorized from lowest to highest severity. To our knowledge, this analysis in colorectal cancer surgery has not been reported previously in the literature.

However, this association has been indirectly reported in pancreatic cancer surgery. In 2022, Bonsdorff et al. [21] studied the occurrence of pancreatitis following cephalic duodenopancreatectomy and demonstrated that patients who developed pancreatitis with elevated CRP had higher CCI scores compared to those with no CRP elevation, indicating that the complications were more severe.

Our team believes that the comprehensive complication index (CCI), which is based in Clavien-Dindo classification, allows for a specific assessment of the overall severity of all postoperative complications.

Subgroup analyses of CRP and complications have already been performed with the Clavien-Dindo classification, and the results have been similar. In a 2015 study of 241 patients, McSorley et al. [8] compared CRP levels on the second, third, and fourth days with the Clavien-Dindo classification, dividing patients into those with no complications (grade 0), mild complications (grades 1–2), or severe complications (grades 3–5). The mean CRP on the fourth postoperative day was 98 mg/L for patients without complications, 161 mg/L for those with mild complications, and 243 mg/L for those with severe complications, with statistically significant results.

We emphasize that CRP-4POD levels in the subgroup of patients who underwent colorectal cancer surgery excluding those with dehiscence were also statistically significantly related to the occurrence of any type of complication. However, the cutoff point to detect these complications was lower. These data support the hypothesis that CRP is a useful parameter for predicting any type of complication occurring in the postoperative period, not just dehiscence.

Platt et al. [22] divided a series of 454 patients undergoing colorectal cancer surgery into three groups: patients without postoperative complications, patients with infectious complications (including anastomotic dehiscence), and patients with non-infectious complications. It was found that CRP was higher in both complication groups compared to the no complication group, but statistical significance was only observed in the infectious complications group. In our study, which did not analyze infectious complications as a separate group, significant differences were found between the no complication group and both the dehiscence and non-dehiscence complication groups. Significant differences in CRP-4POD levels were also observed between the groups with and without dehiscence complications. We believe that an inflammatory state is not always necessarily associated with infection. Any postoperative alteration could lead to an increase in CRP, as seen with cardiovascular problems [23] and acute kidney failure [24].

Various CRP cutoff points have been established for detecting anastomotic dehiscence. Recent meta-analyses have reported a CRP value on the fourth day of 114 mg/L [25] and 123 mg/L [7]. The EDEN Group’s multicenter study describes a cutoff of 119 mg/L with a negative predictive value (NPV) of 97% [19]. In our sample, patients with dehiscence had CRP-4POD levels of 167.6 mg/L. Based on our results, we can state with a high probability (NPV, 81.9%) that patients with CRP < 58 mg/L on the fourth postoperative day will not have postoperative complications and will have a CCI score of 0. However, there is little consensus on the exact CRP value at which we can confidently exclude any type of postoperative complication.

Somewhat higher values have been described by Jin et al. [9] in their series of 335 patients, where a cut-off of CRP > 64.7 mg/L was established for detecting a high CCI score.

The optimal day for CRP measurement in predicting complications remains a subject of debate, since an early assessment with elevated values may lead to a false-negative result in complementary tests [26], while a late assessment may result in an avoidable diagnostic delay. In this study, it was determined on the fourth postoperative day, in line with existing evidence for detecting anastomotic dehiscence on the third or fourth postoperative day, with no significant differences between studies published over the years [7, 16, 27]. However, these studies were focused on detecting anastomotic dehiscence. Regarding the detection of overall complications, it is suggested that CRP should be measured on the fourth day [28, 29], though we have not found studies comparing the third and fourth postoperative days. McSorley et al. [8] compared CRP measurements on the second, third, and fourth postoperative days. They concluded that all days were useful for predicting the severity of postoperative complications but did not analyze which day was optimal for measurement.

In our study, preoperative CRP was not routinely measured. However, a recent systematic review and meta-analysis of 23 studies involving 7147 patients analyzed whether preoperative CRP levels were related to the occurrence of postoperative complications [30]. It could not be shown that elevated preoperative CRP was associated with anastomotic dehiscence, but it was related to the occurrence of overall complications. The results of this meta-analysis support the theory that changes in the inflammatory state of the body, as measured by CRP, can be useful for predicting postoperative deviations from normality.

Traditionally, the development of complications following colorectal cancer surgery, especially anastomotic dehiscence [31, 32], and the presence of an altered postoperative proinflammatory state [33] have been associated with a worsened oncological prognosis [5]. In contrast, more recent studies conducted by the RectoLeak Study Group [4] suggest that no such association exists. This finding could be explained by an earlier diagnosis and the optimization of therapeutic strategies in recent years. Regarding other postoperative complications and their impact on survival [14], similar outcomes may be expected in the future. As suggested by our study, postoperative CRP levels may be useful for the early diagnosis and management of such complications.

CRP is not the only biomarker that has proven useful in the postoperative monitoring of colorectal cancer. In the PREDICS study [34], both CRP and Procalcitonin (PCT) levels were measured on postoperative days 3 and 5. Similar to our study, the values were compared across three patient groups: those who developed no complications, those who experienced anastomotic leakage, and those who suffered postoperative complications unrelated to anastomotic leakage. The study concluded that CRP levels below 12.5 mg/dL and PCT levels below 2.3 ng/mL on the fifth postoperative day were highly effective in ruling out anastomotic leakage, with a NPV exceeding 98%.

It is plausible that the combined use of multiple biomarkers may be more informative than their isolated assessment. This notion is supported by the iCral Study Group [35], who demonstrated not only that PCT levels greater than 1.01 µg/L on the second postoperative day were superior to CRP in predicting mortality, but also that combining the Dutch Leakage Score [36] with both CRP and PCT improved both the PPV and NPV for the prediction of anastomotic leakage, compared to the combination of the Dutch Leakage Score with CRP alone.

As limitations of this study, we must mention that it is a retrospective, single-center observational study and that no analysis by complication subtype was conducted. However, a strength of this study is that it is a consecutive series of colorectal cancer patients and is one of the first studies to use the CCI scale for analyzing postoperative complications in relation to CRP-4POD. Furthermore, it aimed to analyze all postoperative complications, not just anastomotic dehiscence.

We conclude that CRP levels on the fourth postoperative day are statistically significantly related to the occurrence of any type of postoperative complication, not just anastomotic dehiscence. There is a positive correlation between the CCI score and CRP levels. According to our results, a CRP level < 42 mg/L on the fourth postoperative day allows us to exclude the occurrence of postoperative complications.

## Supplementary information

Below is the link to the electronic supplementary material.

ESM 1 (DOCX 33.7 KB) 

## Data Availability

The datasets generated and/or analyzed during the current study are not publicly available due to ethical and legal restrictions concerning the confidentiality and privacy of human participants, in accordance with the Declaration of Helsinki and the General Data Protection Regulation (EU GDPR). Access to de-identified data may be granted upon reasonable request to the corresponding author, subject to prior approval by the relevant institutional review board and in accordance with applicable data sharing agreements.

## Declarations

### Permission to reproduce material from other sources

No material from other sources was included in this article.
